# Data on different sized particulate matter concentration produced from a construction activity

**DOI:** 10.1016/j.dib.2020.106467

**Published:** 2020-10-30

**Authors:** Daniel Cheriyan, Jae-ho Choi

**Affiliations:** a Dept. of Civil Engineering, Dong-A University, 550Bungil 37, Nakdong-Daero, Saha-Gu, Busan, 49315 South Korea

**Keywords:** Construction dust, Particulate matter, Low-cost dust sensor, Occupational health, Monte Carlo simulation

## Abstract

Particulate matter (PM) exposure is produced during most of the construction activities. The dataset was acquired from an experimental investigation by monitoring PM_10_, PM_2.5_, and PM_1_ concentration produced while building a solid concrete block wall. Alphasense OPC-N2 sensors and Sharp GP2Y1010 sensors were collocated in each of the monitoring stations (MS) to measure the PM concentration. The data was collected at 2 s time interval during the entire 40 min of the activity. The data can be utilized for study PM produced and propagated from construction. Furthermore, the dataset can be used to improve the awareness of the construction professionals about the PM production and exposure during the construction works and refine the current construction practices.

## Specifications Table

**Table:** 

Subject	Civil Engineering
Specific subject area	Construction dust management, construction health and safety
Type of data	Table
	Graph
	Figure
How data were acquired	Systematic experiment was conducted to monitor PM concentration generated from a construction activity. Low-cost dust sensors (Alphasense-OPC N2 and Sharp GP2Y1010) were used to measure the PM concentration.
Data format	RAW
	Analyzed
	Filtered
Parameters for data collection	The horizontal and vertical distance between the sensors and the PM source and the angle between them is considered as parameters to reduce the uncertainty of measurements.
Description of data collection	Data was collected using Alphasense-OPC N2 sensor and Sharp GP2Y1010 sensor. Both the sensors were mounted on a monitoring station and the data samples were collected at 2 second time interval.
Data source location	Dong-A University
	Busan
	South Korea
Data accessibility	RAW data from Mendely, “Data on different sized particulate matter concentration produced from a construction activity” DOI:10.17632/6fd493866k.1. https://data.mendeley.com/datasets/6fd493866k/1
Related research article	The data associated is from D. Cheriyan, J. Choi, Estimation of Particulate Matter Exposure to Construction Workers Using Low-Cost Dust Sensors, Sustainable Cities and Society. (2020) 102197. https://doi.org/10.1016/j.scs.2020.102197

## Value of the Data

• Data set of PM concentration generated from construction activity can be useful while arranging potential control measures to reduce the exposure.
• Professionals and authorities responsible for construction projects can use the data to increase the awareness of PM exposure from construction works.
• Researchers in the area of occupational health and construction management can use the data to develop the existing construction practices further.
• The ratio between PM_10_, PM_2.5_, and PM_1_ during mixing task and solid block wall laying task can be used to investigate the PM exposure and settlement time of different sized construction dust.

## 1 Data Description

The presented data (see [2]) is obtained from a systematic experimental investigation of construction activity (i.e., building a solid concrete block wall). The total average PM concentration produced from the activity is presented in Table 1. The constructed block wall was of size (0.5 × 0.6 m), the total average exposure of different sized particles (i.e., PM10, PM2.5, and PM1) measured by Alphasense OPC-N2 sensor during the mixing task were 6660.03, 356.29, and 64.27 µg/m^3^ and those for the laying task were 1071.30, 175.85, and 49.41 µg/m^3^, respectively by the Alphasense OPC-N2 sensor.

**Table 1:** Total average PM concentration produced during the construction activity [2]

		PM Exposure from Alpha sensor			
Construction tasks	Time	PM_10_ (µg/m^3^)	PM_2.5_ (µg/m^3^)	PM_1_ (µg/m^3^)	Total average concentration (µg/m^3^)
Cement sand mixing task	4 – 20 minutes	6660.03	356.29	64.27	2360.20
Solid concrete block laying task	21–40 minutes	1071.30	175.85	49.41	432.18

The sharp sensor measured the same static and dynamic trends similar to that of Alphasense OPC-N2 sensor, but the PM concentration was much higher than that of the saturation point of sharp sensor (i.e., 350-500 µg/m^3^, [1]). Thus it is not used for further analysis. The data measured by Alphasense OPC-N2 sensor is used for further analysis. The activity was carried out for a duration of 40 min, in which the mixing task was executed for 16 min and laying the solid concrete block wall task was completed in 20 min. The results of a two- stage MCS is shown in Table 2, which shows the PM_10_ exposure of construction workers who are building solid concrete block wall in a working day has an average of 60.00 mg/m^3^ exposure of PM_10_. First using standard latin hypercube sampling PM_10_ exposure to the 10 number of construction workers are attained for 40 min of activity. Considering 6 hours work and 2 h rest time in a working day, 9 cycles of 40 min are generated. It is then used to generate total PM_10_ exposure of a working day.

**Table 2:** Two-stage MCS showing total exposure of construction workers for a working day. *Modified from (Cheriy*an and Choi, 2020)*.*[1].

PM_10_ exposure for first cycle of activity (mg/m^3^)										
No. of Workers	1	2	3	4	5	6	7	8	9	10
PM10 concentration	3.79	4.28	4.16	2.59	8.62	6.88	7.55	7.42	4.63	6.48
Standard deviation	2.94	2.94	2.94	2.94	2.94	2.94	2.94	2.94	2.94	2.94
Two-stage Monte Carlo Simulation method: First stage										
PM_10_ exposure for 6 hours of a working day (9 cycles of 40 minutes)										
No. of cycles\No. of workers	1	2	3	4	5	6	7	8	9	10
1	4.27	8.85	6.57	3.56	9.73	7.22	10.35	6.59	3.78	9.95
2	6.66	7.72	10.71	2.21	17.63	15.18	18.72	15.66	14.93	20.90
3	8.68	4.76	14.09	4.54	27.15	19.45	35.96	24.13	20.39	23.31
4	14.43	2.11	17.73	5.13	34.78	19.74	42.02	34.17	28.68	29.54
5	15.51	7.86	22.74	10.02	42.53	26.38	53.04	43.85	36.49	33.41
6	16.74	13.39	27.04	14.01	46.00	34.25	60.85	51.00	34.78	41.19
7	17.00	20.75	28.03	10.79	61.12	39.96	69.45	58.19	39.24	55.11
8	17.29	26.47	29.97	17.65	68.32	46.42	81.69	67.12	43.99	68.49
9	20.62	32.42	31.14	18.31	78.22	51.53	91.51	72.27	52.86	78.31
Total PM exposure	23.95	38.37	32.30	18.97	88.12	56.65	101.33	77.43	61.74	88.13
Two-stage Monte Carlo Simulation method: second stage										
PM_10_ concentration after 1000 simulations of total PM_10_ exposure (mg/m^3^)										
Average	60.53	60.37	60.24	59.59	59.69	60.14	60.72	60.23	58.95	59.83
Minimum	25.62	22.45	23.89	24.65	21.60	24.43	23.86	22.08	21.62	22.25
Maximum	81.85	84.21	81.59	88.05	84.64	81.94	83.83	81.25	82.12	84.83

The total exposure to PM10 attained from 9 cycles is then simulated 1000 times for each worker, the average, minimum and the maximum exposure generated from those simulations are presented in the Table 2.

## 2 Experimental Design, Materials and Methods

The experiment was conducted in an experimental room (from now on dust chamber). The dust chamber is of size 6 × 6 m, which is separated into two (i.e., two numbers of 6 × 3 m). One of the 6 × 3 m area of the dust chamber is used to execute the construction activity, and the other was used to store materials, tools and computer systems. The execution of the dust chamber consists of three monitoring stations (MSs), each MS is collocated with an Alphasense OPC-N2 sensor and a Sharp GP2Y1010 sensor.

**Fig. 1:**
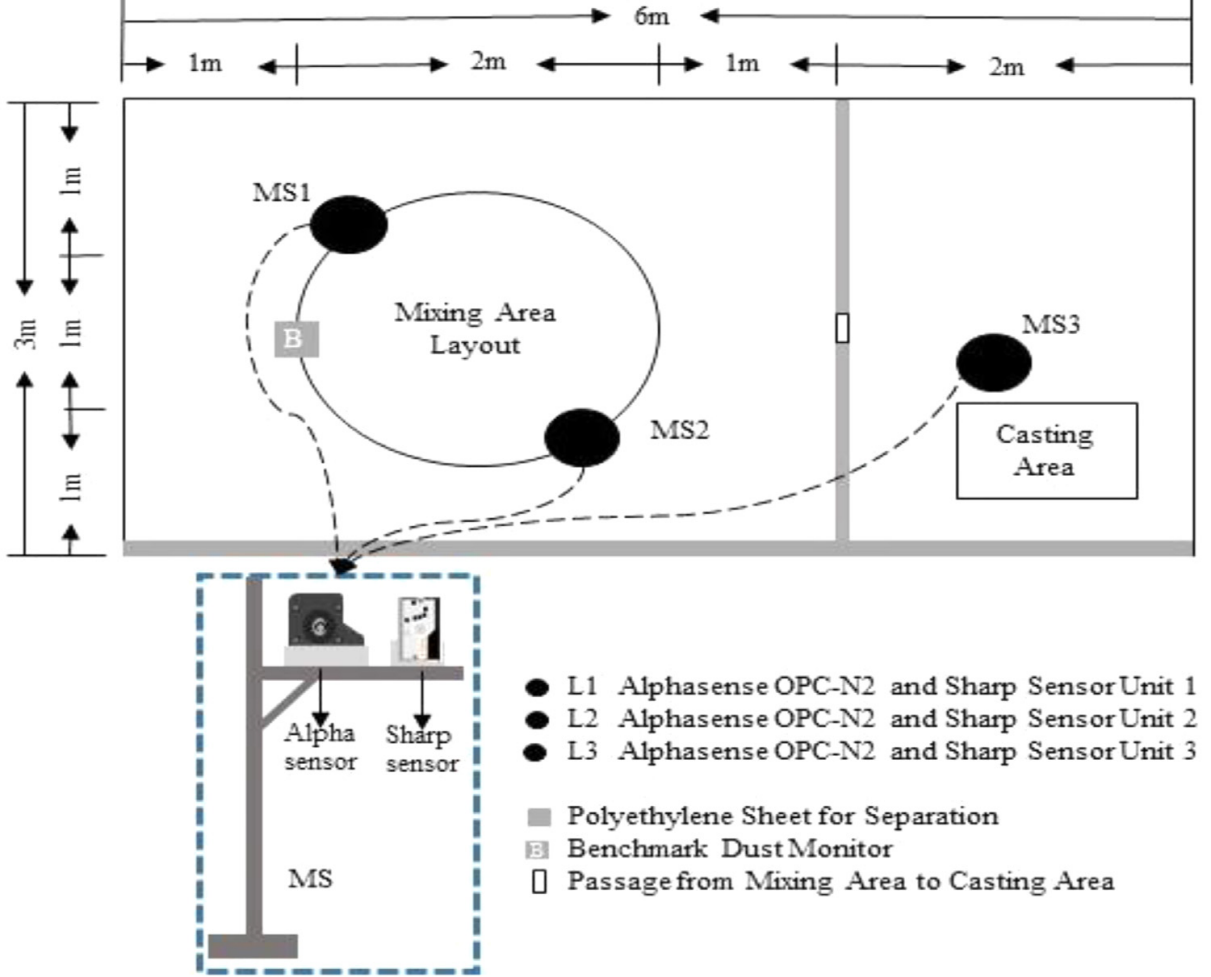
Experimental setup used for measuring PM exposure during a construction activity.

The MS is kept at 1 m horizontal distance from the PM source and 0.8 m vertical distance from the ground level. This distance is kept accommodating the needed working space and the breathing zone of a construction worker while executing the tasks. The activity was conducted as two tasks (i.e., mixing and laying task), mixing and laying tasks are carried out at the allocated areas, respectively (see Fig. 1). Materials used in this experimental study include 65 numbers of solid concrete blocks of size 0.1 × 0.05 m, 7.5 kg of ordinary Portland cement, and 17.7 kg of fine aggregates. The tools used for the execution of the tasks are 1) A shovel, 2) A trowel, 3) A conveying bucket, 4) compacting rod.

## Declaration of Competing Interest

The authors declare that they have no known competing financial interests or personal relationships which have or could be perceived to have influenced the work reported in this article.

## Appendix Supplementary materials

Image, application 1
