# Supplementary material for: Data on different sized particulate matter concentration produced from a construction activity
Source: Data Brief. 2020 Oct 30;33:106467. doi: 10.1016/j.dib.2020.106467 (PMC7644872; doi:10.1016/j.dib.2020.106467)
Supplement: Supplementary file 1 [file mmc1.pdf]

Sustainable Cities and Society 59 (2020) 102197

Contents lists available at ScienceDirect

Sustainable Cities and Society

journal homepage: www.elsevier.com/locate/scs

Estimation of particulate matter exposure to construction workers using low-cost dust sensors

Daniel Cheriyan[a], Jae-ho Choi[b,*]

[a] Dong-A Univ., Dept. of Civil Engrg., P4401-1, 550 Bungil 37, Nakdong-Daero, Saha-Gu, Busan, 49315, Republic of Korea
[b] Dong-A Univ., Dept. of Civil Engrg., P4401, 550 Bungil 37, Nakdong-Daero, Saha-Gu, Busan, 49315, Republic of Korea

A R T I C L E I N F O

Keywords:
Particulate matter
Health effects
Construction dust
Low-cost sensors
Monte Carlo simulation

A B S T R A C T

The construction industry is one of the significant sources which pollutes the environment. As 66 % of the world's population will move towards urban areas within 2050, the rate of construction work happening in the cities will be enormous. Thus, construction practitioners should be aware of Particulate matter (PM) exposure and associated health impacts on society living in urban areas and focus on executing targeted countermeasures. Low-cost sensors with higher sensitivity and higher saturation point can be used for PM measurements during the construction works. This study measures PM produced from a construction activity using low-cost sensors. A total of 4635 exposure samples were collected during the experiment from which the PM concentrations for PM10, PM2.5, and PM1 were recorded as 7731.32, 532.14, and 113.68 μg/m^3 with a standard deviation (σ) of 1990.39, 64.97, and 12.06 μg/m^3. The two-stage Monte Carlo simulation (MCS) method supports the conclusion that even after implementing efficient control measures, the concentration of PM10 and PM2.5 remain 30 and 8 fold higher than their respective 24-hs exposure standards. Thus, future research work should focus on creating targeted control measures, further research exploring the distribution of particles produced from different construction activities should be monitored.

1. Introduction

Air quality is becoming an area of concern for researchers as the environment is being polluted from multiple sources. The construction industry is one of the significant sources which pollutes the environment and endangers human health (Li et al., 2019; USEPA, 1999). Thus, the construction industry poses a threat not only to those who work in it but also to the surrounding environment and the communities associated with it (Azarmi et al., 2016). A detailed study about pollutants emitted by the construction industry has to be conducted in order to counter the occupational hazard and public health concerns generated from the construction activities (Beaudry et al., 2013; Wu et al., 2016). PM (i.e., the total suspended solids and liquid particles in the air) emission is one of the major air pollutants emitted from the construction sites (USEPA, 1999). The exposure to PM_{10} (particles which has a diameter of 10 μm or less), $PM_{2.5}$ (particles which has a diameter of 2.5 μm or less), and PM_1 (particles which has a diameter of 1 μm or less) is extremely harmful to human beings, as it creates critical health issues such as silicosis, cancer, premature death, and asthma (Apte et al., 2018; HSE EH40/2005 Workplace exposure limits, 2018). A study carried out by Peters et al. (2009) briefed that exposure to cement dust is a primary reason for cancer among construction workers. As 66 % of the world's population will be moving towards urban areas within 2050 the rate of construction work happening in the cities will be enormous (Oliveira et al., 2019; Tian et al., 2019; United Nations, 2014). Thus, construction practitioners should be aware of PM exposure and its negative health impacts on society living in that city and focus on executing much-targeted control measures. This can help in creating a sustainable construction practice to keep the current society living in the cities on the safe side.

Although the construction industry is one of the major contributors to air pollution, research work exploring PM production and discharge from the construction activities are still at an early stage (Cheriyan & Choi, 2020). While assessing the research areas related to PM, it is comprehended that researchers are exploring the production of PM_{10}, $PM_{2.5}$, and PM_1 exposure in a broad manner (Fisher et al., 2019; Tian et al., 2019). Research work pertaining to PM exposure is progressing in the aspect like the environmental quality of human beings (Curto et al., 2018). Curto et al. (2018) explored the air quality of households using two low-cost sensors (i.e., HAPEX and TZOA-R) for monitoring $PM_{2.5}$ concentration. They found that the correlation was moderate-high (0.66 − 0.89) between the low-cost sensors, thus, it can be used as a

* Corresponding author.
E-mail addresses: daniel@donga.ac.kr (D. Cheriyan), jaehochoi@dau.ac.kr (J.-h. Choi).

https://doi.org/10.1016/j.scs.2020.102197
Received 8 July 2019; Received in revised form 8 April 2020; Accepted 8 April 2020
Available online 19 April 2020
2210-6707/ © 2020 Elsevier Ltd. All rights reserved.

viable PM monitoring device.

The two leading technological platforms used to monitor the PM (Hall et al., 2014) are USEPA-Federal Reference Method (FRM) and USEPA-Federal Equivalent Method (FEM). In FRM, the monitoring device is positioned to gauge the PM concentration, this method is also known as gravimetric approach. It collects the sample (duration varies from days to weeks) and the sample goes through a series of laboratory experiments to find out the concentration and particle size distribution (EN 12341, 1999; EN14907, 2005). On the other hand, FEM is the modern method in which dust can be monitored in real-time and in this way devices or monitors provide real-time PM concentration data within the defined periods. These devices can provide details like average exposure, peak exposure and particle count (EPA Air Sensor Guidebook, 2014). Dust sensors are considered as a viable device to monitor and measure PM. United States Environmental Protection Agency (USEPA) is a governmental organization that works for environmental protection in the nation. USEPA shows that any sensor which is priced below 2000 USD is a low-cost sensor. Thus, the author considers any PM monitoring device with a price ranging above 2000 USD to 7500 USD as a medium-cost device. The reference PM monitors work on the standard gravimetric principle. The majority of low-cost and medium-cost devices utilize the light-scattering principle. Kanomax 3443 is a commercially available medium-cost PM_{10} monitor which was collated with the low-cost sensors to compare their performance.

Wireless sensor network (WSN) is one among the modern technologies for monitoring a wide range of environmental pollutants at a lower cost. Khadem and Sgarciu (2014) deployed a WSN to collect PM data from low-cost dust sensors. The communication system can operate on a set of AA batteries or from the direct power supply, providing a reliable low power measurement system. Research areas associated with the environmental sciences, healthcare, and smart homes are utilizing the smart sensors to monitor the air quality in the least expensive way (Awolusi et al., 2018).

Budde et al. (2013) and Cao et al. (2013) stated that low-cost dust sensors can be utilized to monitor and gauge PM as it is a modern technology which provides many benefits such as; small size making it easy to handle and deploy, lower cost compared to benchmark monitors (optical photometer), and high correlation of measurements (more than 90 %) with benchmark monitor. Despite the benefits mentioned above, the low-cost dust sensors are not entirely adapted to monitor the air quality at the construction site. Some organizations have conducted experiments to evaluate the performance of dust sensor for both indoor and outdoor conditions, such as USEPA (US EPA, 2018), South Coast Air Quality Management District (SCAQMD, 2018) and the community robotics (The Community Robotics, 2019). They showed that the low-cost dust sensor has a high correlation with the benchmark dust sensor. Many previous authors have highlighted the lack of an informational database concerning the dust emission from the construction activities (Muleski et al., 2005; Tong et al., 2018).

A limited number of research publications are available, highlighting the measurement of PM emission from the construction activities using low-cost dust sensors. Naticchia et al. (2014) monitored the emission of PM from a construction activity (mixing of cement and aggregates) by using the Sharp dust sensor as a low-cost dust monitor. An experimental evaluation to monitor the PM emission from concrete work was conducted by Liu et al. (2016) using low-cost dust sensors. They concluded that low-cost dust sensors show a significant correlation with the benchmark devices. Li et al. (2018) recorded dust exposure using a Sharp dust sensor and SidePak TSI during the execution of various construction activities such as sawing, drilling, and sanding by types of equipment with local exhaust ventilation (LEV) as a control measure. However, the exposure of $2.114\,mg/m^3$ (highest) to $0.127\,mg/m^3$ (lowest) were gauged, which is unhealthy for human inhalation.

Recent research showcases that smaller particles like micro and fine particles (i.e., PM_1 and $PM_{2.5}$) have different properties compared to the respirable particle concentration (PM_{10}) which makes it much more hazardous than PM_{10} particles (Tian et al., 2019). The authors will measure different sizes of PM (PM_{10}, $PM_{2.5}$, and PM_1) emitted from construction activity. After assessing multiple research works in this particular field, the authors found that Alphasense OPC-N2 (hereafter Alphasense sensor) is the only sensor in the low-cost sensor category (costs 500 USD) which records different sizes of PM (i.e., PM_{10}, $PM_{2.5}$, and PM_1) simultaneously (Alphasense OPC-N2, 2015). A report from the World Meteorological Organisation highlights the Alphasense OPC-N2 sensors as a potential device to monitor PM (Lewis, Schneidemsser, & Peltier, 2018). However, many researchers have used a Sharp dust sensor GP2Y1010 (hereafter sharp sensor) which costs around 10 USD for sensors and including all the accessories it will be around 50 USD. Sharp dust sensor measures the total dust density once it is calibrated. Thus the authors will be using Alphasense and Sharp sensor for monitoring PM along with Kanomax PM_{10} monitor.

It is understood that PM exposure generated from different construction activities is still unknown. Quantification of PM exposure produced from the construction activities can play a vital role in resolving the occupational health hazards posed by the construction industry. Monitoring and measuring the PM concentration generated from construction activity is a difficult task, considering the complex nature of the construction works.

This investigation utilizes the dust sensors to broaden the information concerning the production of PM (i.e., PM_{10}, $PM_{2.5}$, and PM_1) concentration from construction activity. Building a solid concrete block wall was selected as an activity to measure the produced PM concentration. Construction of a solid concrete block wall is a common activity at the construction projects, which can be divided into separate tasks to measure the PM emission. The authors have monitored and gauged the PM concentration emitted during the multiple tasks of the selected construction activity. The measurements were recorded by installing the monitoring station (MS) at different locations, considering the key parameters associated with PM monitoring while using dust sensors.

2. Methodology

2.1. Scope

A systematic experimental assessment can bring forth the lacking set of information highlighting the key factors affecting PM emission by utilizing low-cost dust sensors. The steps followed during the experimental investigation is shown in Fig. 1. Construction of a solid concrete block wall was selected to monitor and measure the production and discharge of PM.

While using sensors as PM monitoring device there are parameters to be considered (resuspension and deflection of particles) to reduce the uncertainties associated with the recorded PM measurements. The authors used a moisturized adhesive floor mat to retain the settling particles to reduce resuspension. Along with that to reduce the particle deflection the authors kept 1 m distance between the outlet of the sensors and the boundary wall of the laboratory. The experimental setup and the execution are further detailed in Section 2.3.3. Finally, the PM exposure level of construction workers in the activity was estimated using the stochastic data of the PM concentration obtained from the experiment.

The main aim of this article is to monitor the emission and exposure of PM from a construction activity using low-cost dust sensors such as Sharp and Alphasense. In the experimental setup (see Fig. 2), the MS were arranged such that it accommodates the key parameters which can affect the accuracy of the PM measurements while using the sensors as monitoring device (i.e. deflection and resuspension of PM). The dimension of the experimental laboratory is 6 m × 6 m in length and width, in which 6 m × 3 m of the area is used to execute the experimental activity.

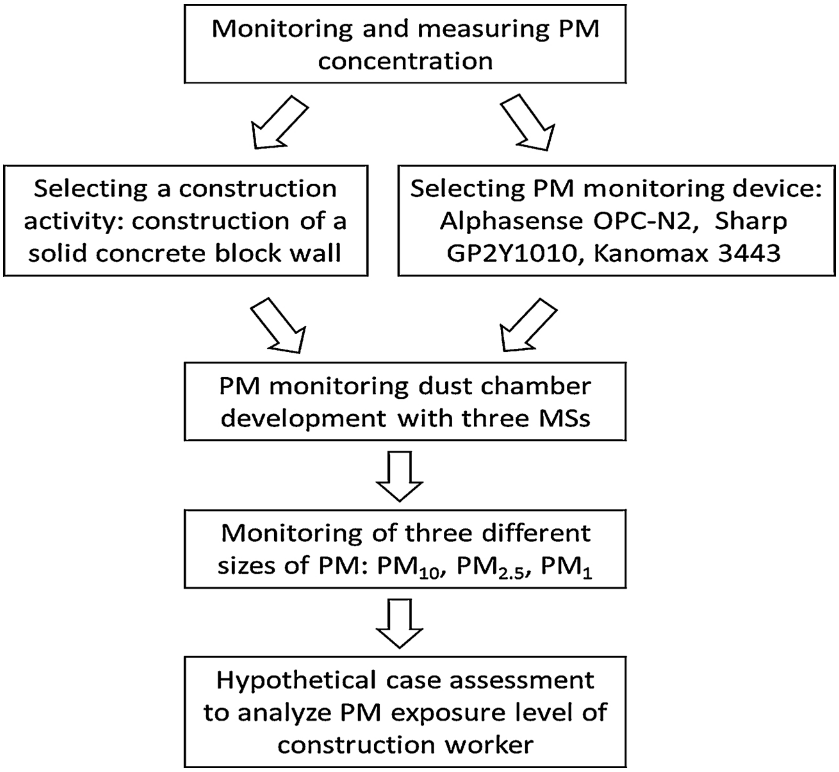

Fig. 1. Methodology to monitor and analyze PM generation from the construction activity.

The mixing and laying area was separated by a polythene sheet while the remaining area of the lab (6 m × 3 m) was used to store materials, tools, and computer systems to record the data from the sensors. Three MSs were installed in the laboratory, at different locations (L1, L2, and L3, see Fig. 2). Each MS contained an Alphasense sensor and a Sharp, the Alphasense sensors and the Sharp sensors are named as A1, A2, and A3 and D1, D2, and D3, respectively at the three MSs.

2.2. Materials

The authors constructed a solid concrete block wall with class 2 mortar as an experimental model of size (0.5 m × 0.6 m × 0.09 m). Materials and tools used in the execution were 1) Number of the solid concrete block of size (0.10 m × 0.05 m × 0.09 m) = 65 Nos, 2) Amount of Ordinary Portland cement used = 7.5 kg, 3) Amount of fine aggregates used = 17.71 kg.

Note: the material showcased above is after allocating 5% wastage for mortar and a 2.5 % wastage for concrete blocks.

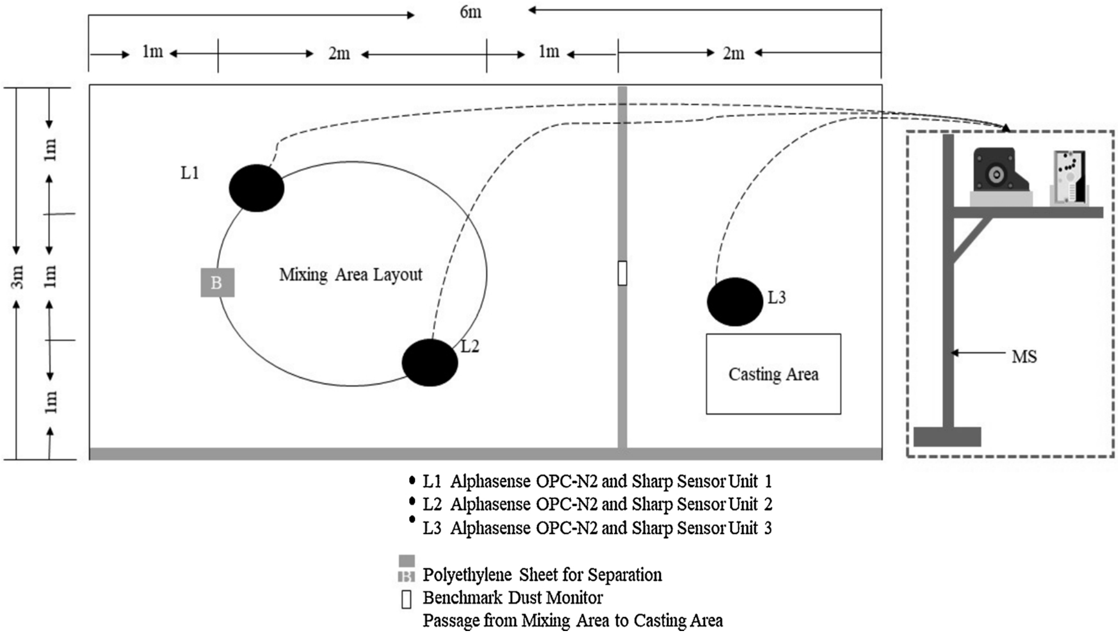

Fig. 2. Experimental setup to monitor PM emission from a construction activity.

Table 1
Comparing the specification of the Sharp and Alphasense sensor.

Specification	Sharp Sensor GP2Y1010	Alphasense OPC-N2
Particle Range (μm)	–	0.38–17
Particle size (μm/m^3)	Dust density	PM_{10}, $PM_{2.5}$, and PM_1
Sampling Interval (Seconds)	1	1–30
Max. Particle Count Rate	NA	10,000
Data Storage	NA	Micro SD
Temperature Range (°C)	−10 to 65	−20 to 50
Humidity Range (% RH)	95	99
Weight (g)	25	105
Usage	Static/Movable	Static/Movable
	Sharp Corporation (2006).	Alphasense OPC-N2 (2015)

2.3. Monitoring and measuring PM

In the experimental setup, the dust sensors (i.e., Sharp sensors and Alphasense sensors) were chosen based on the recommendations made by previous researchers (Lewis et al., 2018; Patel et al., 2016; Sousan et al., 2017). Both the sensors work on the light scattering principle, which is widely used as it provides a higher correlation with other PM monitoring devices working on principles such as FRM (Webster, 1999).

2.3.1. Alphasense and sharp sensors

Alphasense sensors can be used for both static and dynamic PM monitoring, and their further specification is as shown in Table 1. The recorded data can be directly transferred from Alphasense to Computer through the USB port. Alphasense sensor is among the low-cost dust sensors which are listed USEPA. The right side of Fig. 3 illustrates the physical state of the Alphasense sensor and its connection. Alphasense sensor sensors are connected to a personal computer with its user interface software which decodes the measured data from the sensors. Apart from this, the Alphasense sensor has many benefits over the Sharp sensor, such as a storage secure digital card to record data and built-in software for the easy user interface. Alphasense sensor has a higher sensitivity towards lower PM concentrations (i.e., 0.38–17 μm). It monitors and measures the concentration of different sizes of PM {i.e., PM_{10} ($PM_{10-2.6}$), $PM_{2.5}$ ($PM_{2.5-1.1}$), and PM_1 ($PM_{1-0.38}$) (Alphasense OPC-N2, 2015)}.

A Sharp dust sensor is an economical option among the selected dust sensors, which cost less than most of the other dust sensors. For monitoring PM concentration, the Sharp dust sensor requires interfacing with an external microcontroller and display (Naticchia et al., 2014). The least sampling time can be defined as 1-second for data recording and transferring from the end-user device to a master node. To attain accurate readings, it is preferred to define a minimum time interval for recording the sample. It has low weight and can be used for both static and dynamic use cases. The design circuit consists of three Sharp sensors (D1, D2, and D3) along with an Arduino UNO Board, temperature and humidity sensor, and Bluetooth for transferring data from the slave node to the master node through wireless networking system as shown in the left side of Fig. 3 (Sharp Corporation, 2006). Sharp sensor monitors and measures dust density at 1-second time interval. It shows a higher correlation with standard benchmarking PM monitors when calibrated with a temperature and humidity sensor (Budde et al., 2013; Sharp Corporation, 2006). The sharp sensor gives output in voltage, this output was then corrected using a offset correction. After which the sensor was calibrated with the equation "dust density (mg/m^3) = ΔV/K) × 100". Where ΔV = Vout - Voc, Voc is the output with no dust, Vout is the output by measuring dust, and K is the sensitivity.

2.3.2. Correlation and Precision of low-cost sensors Alphasense and Sharp sensors

Before the experiment, Four Alphasense sensors and four Sharp sensors were assessed, in which the fourth sensor of both sensor types acted as a standby considering the possibility of any errors or malfunctioning of sensors. Four number of Alphasense sensors (A1, A2, A3, and A4) and four Sharp sensors were operated for 19-hs (65208 sampling points, at 1-second interval period). To increase the correlation between sensors, smoothing of the graph was done by using the moving averages of different time periods (i.e., 1, 5, 30, and 60-seconds). The results showed that the increase in the average period resulted in a higher correlation. The same measured data of each sensors were used

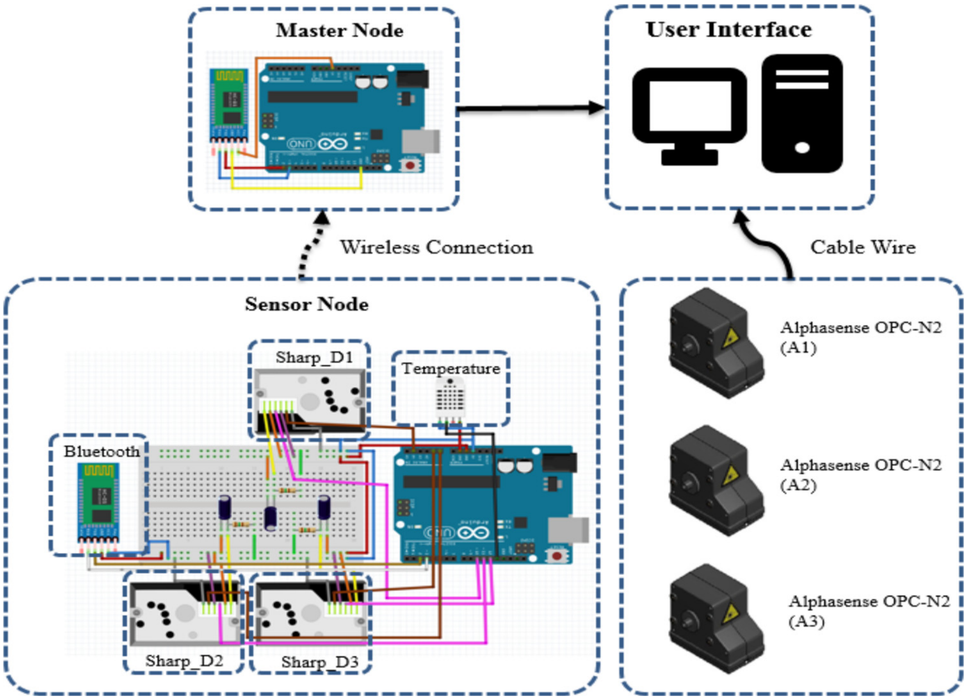

Fig. 3. Connection of Sharp and Alphasense sensors.

to calculate the precision among the Alphasense sensors.

The sharp sensor was also installed beside the Alphasense sensor, but it did not measure any dust particles. It has low sensitivity towards lower dust concentration (below 50 μg/m^3 or 0.05 mg/m^3), as stated in previous articles (Liu et al., 2016; Olivares & Edwards, 2015). Whereas, the Alphasense sensor recorded the peak value of 30 μg/m^3 (0.03 mg/m^3); therefore, response to low PM concentration is recorded by the Alphasense sensor, but not by the Sharp sensors.

2.3.3. Execution of experiment

To analyze the PM concentration generated from construction activity, the authors constructed a solid block wall of size 0.5 m × 0.6 m × 0.02 m. The authors conveyed the materials for mixing cement and sand mortar to the mixing area and made necessary arrangements for the activity (arranging the MS) which took 4 min. The execution of the cement and sand mortar mixing took 16 min for completion after which the mortar was used to lay a solid concrete block wall. The authors laid the solid concrete block wall in 20 min. The total duration of the experiment was 40-min.

3. Results

3.1. Results of correlation and precision assessment of low-cost sensors

The data recorded by the Alphasense sensor at 1-second sampling time showed a correlation of 0.80 for PM_1, and $PM_{2.5}$ while for PM_{10} 0.37 was calculated. After smoothing the curves by taking the 5-second average, a 0.95 correlation was recorded for PM_1 and $PM_{2.5}$ whereas for PM_{10} it was 0.76. A similar enhancement was recorded for 30 and 60-second average interval period. At a 60-second average time interval, more than 0.96 correlation was observed between the Alphasense sensor sensors for all PM sizes.

The precision of all the four Alphasense sensors was checked using the recorded data. The results showed that the recorded PM concentration of all the four sensors was close to each other. Considering the precision of each sensor with the mean of all four sensors A1, A2, A3, and A4 showed 99.80 %, 99.78 %, 99.74 %, and 99.93 %, respectively.

3.2. Experimental results

The average concentration of PM_{10}, $PM_{2.5}$, and PM_1 measured by Alphasense sensor during the mortar mixing task were 6660.03, 356.29, and 64.27 μg/m^3, respectively. The total average concentration from the mixing task was recorded as 2360.20 μg/m^3 from the Alphasense sensor, as shown in Table 2.

The average concentration of PM_{10}, $PM_{2.5}$, and PM_1 measured by Alphasense sensor during the solid block wall laying task were 1071.30, 175.85, and 49.41 μg/m^3, respectively. The total average exposure from the block wall laying task was recorded as 432.18 μg/m^3 by Alphasense sensor (see Table 3).

After monitoring the PM concentration on 2-second interval during the entire activity and assessing it, the authors found that the PM exposure was often significantly higher than that of the PM exposure standard set by the world health organization (WHO) (20 μg/m^3 PM_{10} annual average and 50 μg/m^3 PM_{10} 24-hs average, (WHO, 2005)). Peak PM_{10} concentration measured by the Sharp sensors D1 and D2 at location L1 and L2 (mixing area) was 350 μg/m^3 and 300 μg/m^3, respectively (see Fig. 4(a)). The Sharp sensor D3 located at L3 (block laying area) recorded a maximum peak value of 190 μg/m^3 PM_{10} exposure. The peak measurement recorded by the Kanomax PM_{10} monitor was 425.5 μg/m^3, as shown in Fig. 4(a).

OSHA (occupational safety and health administration of the U.S.A) occupational PM exposure standard for 8-hs and the WHO's 24 is 0.05 mg/m^3 (OSHA, 2018). During 40 min of activity, 95 % of the time, the exposure to PM was above the OSHA occupational exposure standard and WHO's 24-hs exposure standard. The average PM_{10} exposure measured by the Sharp dust sensor and the Kanomax PM_{10} monitor showed the peak exposure as 250 μg/m^3 and 280 μg/m^3 and lowest PM concentration as 100 μg/m^3 and 140 μg/m^3, respectively (see Fig. 4(a)). Fig. 4(b) illustrates the PM_{10} measurements taken by the Alphasense sensor, in which it is visible that the PM concentration was above the exposure standards.

In Fig. 4(b), WHO's the annual exposure level of PM_{10} is not separately visible as the figure is in a 1:1000 scale. The PM concentration was continually increasing during the cement and sand mortar mixing task and approached the peak point prior to the start of the wet mixing (see Fig. 5(a)). The final round of the dry mixing ended by 9-min of the activity which approximately coincides with the settlement time of the PM_{10} sized particles (Baron, 2010). Thus the PM_{10} concertation decreases gradually after 9 min of the construction activity (see Fig. 4(a) and (b)).

The average exposure from different MSs for 16-min duration (i.e., during cement-sand mixing activity) peaked above 6000 μg/m^3, and remained around 2000 μg/m^3 even during the last stage of the activity. Thus, it is understood that PM exposure was above the air quality standards proposed by the OSHA and WHO during the entire activity. Even though the sensors are placed at the same distance from the mixing area, the A1 sensor showed more fluctuation than that of sensor A2, as shown in Fig. 5(a). During the mixing task, the materials moved closer to sensor A1, creating a higher particle propagation towards the A1 sensor. Mixing of cement and fine aggregate was carried out six times (three with pouring water and three without, time for each round of mixing is illustrated with a vertical line in Fig. 5(a)).

Initially, the materials were conveyed to the middle of the mixing area which was equidistant from both the MS. While mixing, two out of six times it was closer to sensor A1 and one out of six times it was closer to sensor A2, and the balance three were carried out at the middle. The sensor unit A3 has lesser exposure during mixing activity as the sensor is placed in the block laying area (which was separated by a polyethylene sheet with a lean opening for material conveyance). As the average PM_{10} concentration illustrates, during the dry mixing, it peaked above 6000 μg/m^3 and remained around 4000 μg/m^3 even during the wet mixing (i.e., while pouring water during mixing). Thus, it can be understood that although the usage of premixed bags for mortar can address the time consumption issue, it still has considerably high PM exposure. The PM concentration and particle count produced during the activity is shown in Fig. 5(a) and (b), respectively.

After the completion of the mixing task, PM_{10} remained suspended and then it gradually decreased based on the settling time of different particle sizes (see Fig. 4(b)). Considering the standards set by Health and Safety Commission for Portland cement inhalation as 10 mg/m^3

Table 2
Emission from mixing task measured by Alphasense sensor.

Sensor No.	Time	PM count	PM Exposure			
			PM_{10} (μg/m^3)	$PM_{2.5}$ (μg/m^3)	PM_1 (μg/m^3)	Average concentration (μg/m^3)
A1	4–20 min	580,118	5330.20	352.98	68.95	1917.38
A2	4– 20 min	415,874	7989.85	359.60	59.59	2803.01
Total average concentration (μg/m^3)	4– 20 min		6660.03	356.29	64.27	2360.20

Table 3
Emission from laying block wall task measured by Alphasense sensor.

Sensor No.	Time	PM count	PM Exposure			
			PM_{10} (μg/m^3)	$PM_{2.5}$ (μg/m^3)	PM_1 (μg/m^3)	Total average concentration (μg/m^3)
A3	21–40 min	264665	1071.30	175.85	49.41	432.18

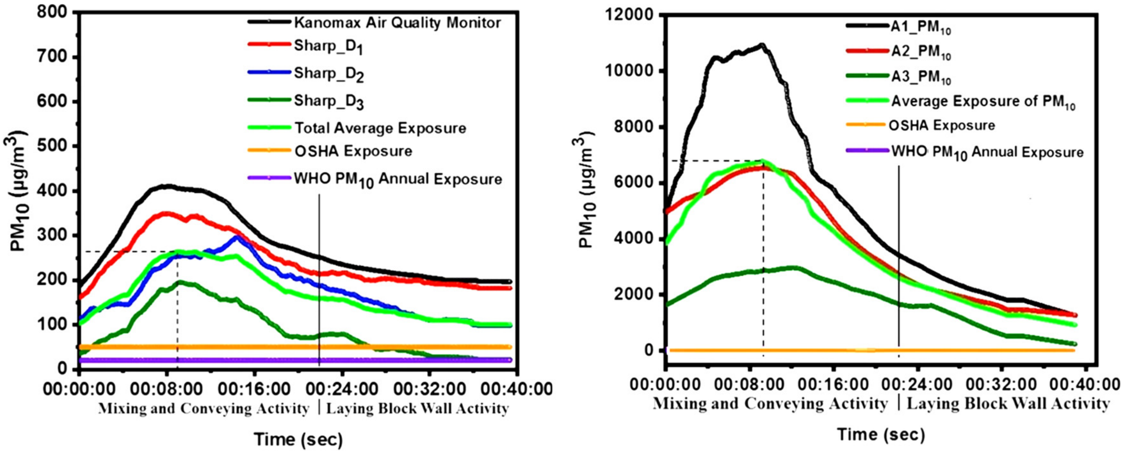

(a) (b)

Fig. 4. (a) PM concentration measured by the Sharp sensor and benchmark, (b) PM_{10} measurements taken by Alphasense sensor.

and respirable as 4 mg/m^3 and for silica (crystalline) inhalable level as 6 mg/m^3 and that for respirable as 2.4 mg/m^3 (Health & Safety Comission, 2005). The exposure to materials used in the task (i.e., Cement and fine aggregate) may be harmful to the individuals executing the activity, as it was higher than the optimum level. $PM_{2.5}$ measurements from the Alphasense sensor showed a hazardous level of PM concentration generated during the activity as showed in Fig. 6(a). The average $PM_{2.5}$ concentration measured by the Alphasense sensors showed the highest above 350 mg/m^3 and the lowest as 200 mg/m^3 (see Fig. 6(a)). Exposure to the fine dust ($PM_{2.5}$) was significantly higher than the optimum exposure levels advocated by the WHO (i.e., 25 mg/m^3). The exposure to fine dust remains above the optimum level throughout the experiment once the mixing task was finished. Thus, the exposure of $PM_{2.5}$ during the erection of a solid concrete block wall was much higher than that produced by that construction task.

The recorded reading from the Alphasense sensor highlights the peak exposure as 400 μg/m^3 within a short duration of the construction activity. The propagation of $PM_{2.5}$ in the working ambiance was quicker than that of PM_{10} as the reading of $PM_{2.5}$ was seen on sensor A3 (Alphasense sensor 3) before the conveying activity. As the settlement time of $PM_{2.5}$ or less sized particles is 12-hs (Baron, 2010), the exposure to $PM_{2.5}$ was not decreasing during the whole time of activity. PM_1 particles propagated in the working ambiance in a rapid manner, which was observed while comparing the recorded measurements of all three Alphasense sensors used during the experiment. A3 sensor shows PM_1 measurements just after 2-min from the initiation of the activity (see Fig. 6(b)).

It is also notable that from the peak PM concentration of (80 μg/m3) 60 % of particles (i.e., 48 μg/m^3) remain suspended in the working ambiance during the entire activity. It is due to the more extended

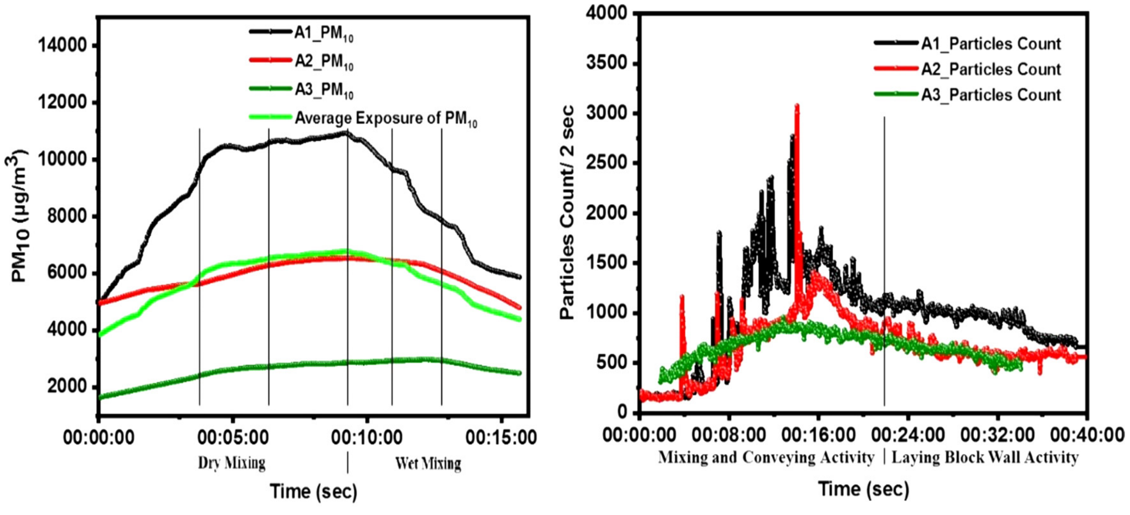

(a) (b)

Fig. 5. (a) PM_{10} measurements taken by Alphasense sensor for mixing task, (b) Particle emission during the entire activity.

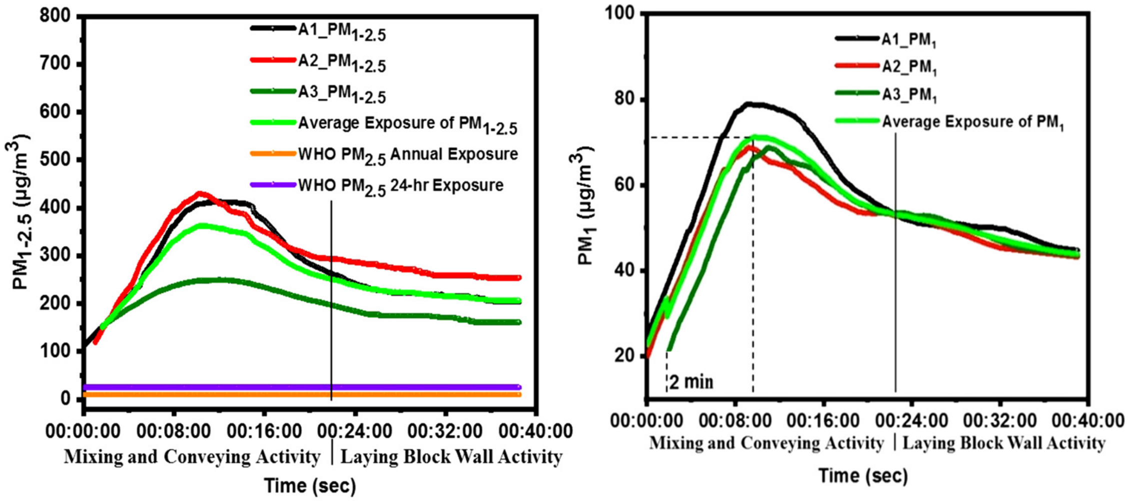

(a) (b)

Fig. 6. (a) $PM_{2.5}$ and (b) PM_1 measurements taken by Alphasense sensors.

settlement time requirement for PM1 particles (Baron, 2010). Considering the real scenario of construction work, the duration of the activity will be more on large scale construction projects which can affect numerous construction practitioners and individuals living in the neighborhood. RCS can be found in construction materials such as sand, stone, concrete, block, and mortar.

According to Stacey et al. (2018) and USEPA (1999), the exposure of RCS may vary between 10 and 2.5–5% of the total average PM_{10} concentration, respectively. Fig. 7 shows the RCS exposure calculated from the experimental construction activity. The area under the curve represents the total average RCS exposure as 0.406, 0.101 and 0.203 mg/m^3 for 10, 2.5 and 5% respectively, which is higher than exposure standards of many countries (GESTIS International Limit Values, 2018).

4. Statistical assessment of results

During the execution of the cement- sand mixing task, the PM concentration was recorded by the MS 1 and 2. A total of 3840 number of samples were monitored during 16-min of the construction task. Sensor A1 recorded the mean PM concentration for PM_{10}, $PM_{2.5}$, and PM_1 as 5330.20, 352.98, and 68.95 µg/m^3 with a σ of 723.23, 57.38, and 9.35 µg/m^3, respectively. The PM concentration measured by sensor A2 was in a similar range except for PM_{10} concentration, which showed particle concentration (see Table 4).

The difference in percentage for $PM_{2.5}$ and PM_1 readings recorded by A1 and A2 (Alphasense sensors) shows 1.8–15.7 %, whereas in

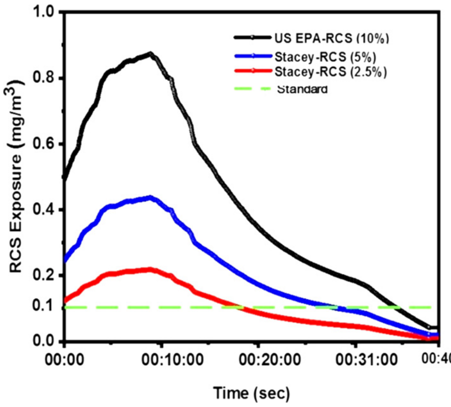

Fig. 7. RCS exposure calculated from the recorded PM_{10} data.

contrast to the particle size PM_{10} the difference between the two sensors was high (i.e., 49.9 %). Sensors D1 and D2 measured PM_{10} concentration along with the A1 and A2, but as the saturation point of the sensors (D1 and D2) were reached in the early stage of the activity the readings were not compared with that of Alphasense sensors. Patel et al. (2016) stated that the Sharp sensors got saturated when the particle concentration is too high (i.e., above 350 – 500 µg/m^3). The same issue was encountered with the D3 sensor during the execution of the following construction task. The Kanomax PM_{10} monitor which measured the PM_{10} particles has a saturation zone of 10 mg/m^3 (Kanomax 3443, 2019), the exposure in the laboratory reached above that zone within the early time period of the experiment. The total dust density measured by the Sharp sensor was lower than the PM_{10} concentration monitored by the Kanomax PM_{10} monitor.

The PM_{10} concentration gauged by those sensors were not plotted in Table 5. Sensor A3 monitored the PM concentration in the block laying area. A total of 1755 samples were taken during the 20-min of the construction task. $PM_{2.5}$ measurements were missing in the recorded data from the time 37 min and 32-seconds to 40-min. Thus, the number of samples taken for $PM_{2.5}$ was 555 (see Table 5). Interpreting the total number of measured PM samples (4635) the authors calculated the time-weighted average (TWA) for 8 -hs to compare the exposure with occupational health standards. The TWA for PM_{10} calculated was much higher than the mean PM concentration shown in Tables 4 and 5. The OSHA standard for 8 -h exposure to respirable dust in a working day is 0.05 mg/m^3. The calculated TWA for PM_{10} exposure was 96.78 mg/m^3 without any control measures. This is calculated considering 6 -hs of work time (9 cycles of 40-min) and 2 -hs rest time (OSHA standard 0.05 mg/m^3 was considered). If implemented with highly efficient control measures (i.e., 80 % of efficiency) then the exposure was reduced to 19.35 for PM_{10}, which is still an unhealthy PM10 exposure level. As OSHA standard is set for the respirable-size fraction (PM_{10}) the authors are utilizing the WHO standard for discussing the fine and microparticles exposure.

The Sharp sensor showed more than 0.90 in correlation with the Alphasense sensors for both the cement-sand mortar mixing task and the laying of the solid block wall. At the same time, the Sharp dust sensor showed more than 0.80 in correlation with the Kanomax PM_{10} monitor as shown in Table 6. The total average PM concentration during both the mixing and laying task for PM_{10}, $PM_{2.5}$, and PM_1 was 7731.32, 532.14, and 113.68 µg/m^3 with a σ of 1990.39, 64.97, and 12.06 µg/m^3, respectively (see Tables 2 and 3). It is recommended to use sensors with a high saturation level to monitor the PM emission from the construction activities, which generates a tremendous amount

Table 4
Statistical assessment of PM concentration recorded during the mixing task.

Desc.	Cement-sand mixing task- statistical analysis								
	Alphasense sensor						Sharp sensor		Kanomax
	PM_{10} (μg/m^3)		$PM_{2.5}$ (μg/m^3)		PM_1 (μg/m^3)		PM (μg/m^3)		PM_{10} (μg/m^3)
	A1	A2	A1	A2	A1	A2	D1	D2	3443
Mean	5330.2	7989.8	352.9	359.6	68.9	59.5	303.5	236.9	331.1
Median	5568.7	8799.7	368.8	360.5	71.5	60.9	314.7	248.6	345.2
Std. dev.	723.2	2213.5	57.3	48.9	9.3	7.1	36.8	37.6	84.7
Min.	3499.8	4152.9	215.1	233.6	44.6	38.4	228.9	144.9	143.5
Max.	6062.6	10433.2	413.1	429.8	78.9	68.7	349.5	295.7	425.5
No. of samples	480	480	480	480	480	480	480	480	480

Table 5
Statistical assessment of PM concentration during the Solid block wall laying task.

Solid block wall laying- statistical analysis			
Desc.	A3 PM_{10} (μg/m^3)	A3 $PM_{2.5}$ (μg/m^3)	A3 PM_1 (μg/m^3)
Mean	1,071.30	175.85	49.41
Std. dev	582.43	12.70	3.84
Minimum	240.26	161.00	43.69
Maximum	2,081.65	206.95	55.87
No of samples	600.00	555.00	600.00

Table 6
Correlation of PM sensors and Kanomax PM10 monitor.

Area in Laboratory	Sensors	Alphasense		Kanomax 3443	
		A1	A2	A3	
Mixing area	Sharp sensor 1	0.9143			0.8216
Mixing area	Sharp sensor 2		0.9041		0.8800
Laying area	Sharp sensor 3			0.9383	0.8000

of PM.

4.1. Hypothetical case assessment

The authors conducted a hypothetical case assessment for a group of construction workers (i.e., 10 workers) assigned to building a solid concrete block wall to assess their PM (mainly to fine and micro-particles) exposure.

As shown in Fig. 8, the authors used standard Latin hypercube sampling to randomly select PM_{10}, $PM_{2.5}$, and PM_1 exposure for each of the 10 workers during the first cycle of activity (i.e., 40-min, see Table 7) using the mean concentration of the three PM sizes (i.e., PM_{10}, $PM_{2.5}$, and PM_1) along with their respective σ ($σ_{PM10}$, $σ_{PM2.5}$, $σ_{PM1}$). This sampling method was used to generate random PM concentrations for each worker in each subset of the cumulative probability distribution range by using the measured PM statistical assessment data, thus artificially making a condition that each construction worker has a different level of PM exposures.

According to Morgan and Henrion (1992), the number of simulation runs required in an MCS to analyze uncertainty can vary from ten to a few hundred. The authors assumed that the workers work 8 -hs a day with 2 -hs' rest time. The 6 h of construction time is divided into 9 cycles, each of 40-min. Two-stage MCS was executed to generate a hypothetical scenario in which the construction workers are being exposed for 6 -hs. In the first stage, 9 simulations are conducted using the PM exposure generated in the first cycle to attain exposure for the selected working day of 6 -hs. The Cumulative PM exposure for each worker during 6 -h work is calculated from the 9 cycles, 40-min each separately for 10 workers (see Tables 8 and 9).

Even if control measures are used, the efficiency of control measures can vary depending on the manner of implementation. Nij et al. (2003) stated that if control measures such as water suppression and LEV can be implemented effectively, then 70–80 % of PM can be reduced. Thus, the authors are utilizing that efficiency as proposed by Nij et al. (2003) as 70, 75, and 80 % (see Tables 8 and 9). The cumulative PM calculated from 9 cycles for the 10 workers is shown in Tables 8 and 9 for both the cement-sand mixing and block laying task along with the reduction in PM exposure while using control measures. From Tables 8 and 9, it is clear that even after assuming 80 % efficiency for control measure, the average PM_{10} and $PM_{2.5}$ exposure remains more than 30 and 8 times the standard exposure level of 50 and 25 μg/m^3 (0.050 and 0.025 mg/m^3) proposed by OSHA and WHO for 24 -hs, respectively (see Tables 8 and 9).

The cumulative exposure of each worker was then simulated 1000 times; thus, a total of 10,000 simulations were obtained for 10 workers. The simulations refined the results much more which can be seen while comparing the average exposure of all the workers before and after the simulations, the average PM_{10} emission from the mixing task before simulation was 55.9 mg/m^3 (see Table 8) and the same after the 1000 simulations is 60.03 mg/m^3 (see Fig. 9(a)). The average PM_{10} emission from the laying task before simulation was 7.6, as shown in Table 9 and the same after the 1000 simulations was 7.4 mg/m^3 (see Fig. 9(b)). Both figures indicate that the Average PM_{10} and $PM_{2.5}$ exposure calculated from the two-stage MCS are well above the standard 24 -hs exposure limit, respectively, even after considering the maximum efficiency of existing control measures (i.e., 11.2, 0.6 and 1.5,0.2 mg/m^3 (see Tables 8 and 9)). An analysis should be conducted for producing a targeted control measure for various construction activities based on their PM dispersion profile. The control measures implemented at construction sites should be a much targeted one, which may vary based on the type of activity and materials and tools used.

5. Discussion and recommendation for further research

The presence of dust particles at the construction sites is being considered as a natural one, against which much awareness is needed to implement effective control measures. The knowledge about the health hazards caused by PM exposure needs to be increased among the construction practitioners to reduce the health issues raised by the PM. To counter these issues, much-targeted control measures should be implemented, for which the PM production and its dispersion from different construction activities need to be understood, which is currently unknown (Cao & Zhang, 2019). The majority of the population will be shifting to urban areas for economic reasons which increase the demand for new construction. Thus the construction projects targeting that market should also consider building a sustainable city to accommodate the future movement of people. This reduces the effect on air pollution

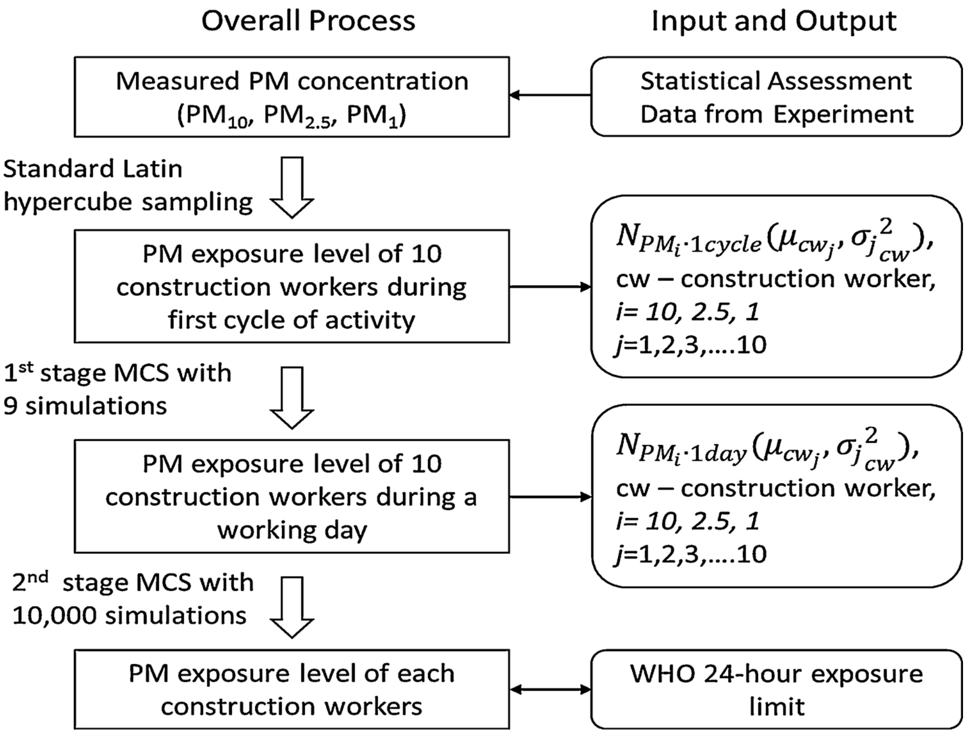

Fig. 8. PM exposure determination using standard Latin hypercube sampling and two-stage MCS.

from the construction and provides the existing society there to live in a cleaner city environment. As the results of this experiment illustrated, a relatively shorter activity (i.e., 40-min) discharged an enormous amount of particles to the breathing zone of individuals during the activity. The optimal PM_{10} exposure limit proposed by WHO for 24-hs and annually are 50 and 20 µg/m^3, respectively. Both the 24-h and annual exposure standard limits were crossed in just 40-min of the executed construction activity.

The PM produced form the cement-sand mixing activity can be suspended in the working ambiance for up to 12-hs (considering the settlement time of fine particles). The average $PM_{2.5}$ concentration recorded by the Alphasense sensors was 356.29 µg/m^3 and the highest measured concentration of $PM_{2.5}$ was 359.60 mg/m^3 during the experimental evaluation. This creates an ambiance in the construction sites which affects not only skilled workers associated with that trade but also other construction practitioners who enter that location within 12-hs. Cheriyan and Choi (2020) showcased that the requirement for real-time monitoring of the activity level PM emission is required and low-cost sensors can play a key role in this aspect. They also pointed out it is important to understand that the PM monitoring for construction works is still at a premature state and distributional characteristics of different sized PM should be investigated to employ appropriate control measures. Thus, educating the construction practitioners about the hazards of PM exposure at construction sites may increase awareness and push towards implementing better control measures. Many previous researchers (Muleski et al., 2005; Tong et al., 2018) pointed out the lack of informational database concerning the PM emission from the construction activities to implement an effective control measure.

Simultaneously monitoring different particle sizes such as PM_{10}, $PM_{2.5}$, and PM_1 in real-time was a complex task, but the complexity was reduced to an extent with the usage of low-cost dust sensors. To minimize the chances of errors (i.e., resuspension and deflection of particles) while using low-cost dust sensors, the authors have used moisturized adhesive floor mats. It can reduce the resuspension of PM during the experimental activity. Otherwise, while the construction workers maneuver in the working area, re-suspension of PM may occur. The authors also have provided enough horizontal distance between the MS and the boundary of the experimental laboratory to reduce the malfunctioning of the PM measurements due to the deflection of particles from the boundary area. The experiment analyzed a smaller construction activity with less size and volume, as the size and volume of the structure increase the PM concentration can vary significantly, which in turn increases the PM exposure of the construction workers.

The 10-min average measurements of PM_{10} from the Sharp dust

Table 7
PM exposure generated from Standard Latin hypercube sampling for the first cycle.

Mean	Number of workers									
	1	2	3	4	5	6	7	8	9	10
	Cement-sand mixing task for block work (mg/m^3)									
PM_{10}	3.79	4.28	4.16	2.59	8.62	6.88	7.55	7.42	4.63	6.48
$PM_{2.5}$	0.41	0.31	0.33	0.34	0.39	0.32	0.23	0.38	0.37	0.31
PM_1	0.07	0.06	0.06	0.06	0.07	0.07	0.06	0.06	0.06	0.06
	Solid block wall laying task (mg/m^3)									
PM_{10}	0.96	0.96	0.99	0.73	0.98	1.01	0.82	0.97	0.96	0.96
$PM_{2.5}$	0.18	0.19	0.20	0.16	0.18	0.18	0.16	0.17	0.14	0.15
PM_1	0.049	0.046	0.061	0.053	0.047	0.041	0.055	0.053	0.046	0.047

Table 8
PM exposure: with and without control measures for mixing task.

% of PM reduction	Without control measures (mg/m³)			With control measures (mg/m³)								
				70 % efficiency			75 % efficiency			80 % efficiency		
Cumulative PM exposure of workers	PM_{10}	$PM_{2.5}$	PM_1	PM_{10}	$PM_{2.5}$	PM_1	PM_{10}	$PM_{2.5}$	PM_1	PM_{10}	$PM_{2.5}$	PM_1
1	45.7	3.8	0.6	13.7	1.1	0.18	11.4	0.9	0.15	9.1	0.7	0.12
2	46.8	2.7	0.5	14.1	0.8	0.16	11.7	0.7	0.13	9.3	0.5	0.10
3	39.1	2.9	0.5	11.7	0.8	0.17	9.7	0.7	0.14	7.8	0.5	0.11
4	16.9	3.0	0.4	5.1	0.9	0.14	4.2	0.7	0.12	3.4	0.6	0.09
5	79.8	3.4	0.6	23.9	1.0	0.18	19.9	0.8	0.15	15.9	0.7	0.12
6	67.1	2.8	0.6	20.1	0.8	0.2	16.7	0.7	0.17	13.4	0.5	0.13
7	81.4	2.0	0.5	24.4	0.6	0.18	20.3	0.5	0.14	16.3	0.4	0.12
8	75.8	3.2	0.5	22.7	0.9	0.16	18.9	0.8	0.13	15.1	0.6	0.10
9	36.7	3.5	0.5	11.1	1.0	0.16	9.18	0.8	0.13	7.3	0.7	0.11
10	69.7	2.9	0.5	20.9	0.8	0.16	17.4	0.7	0.14	13.9	0.5	0.10
Avg. exposure	55.9	3.0	0.5	16.7	0.9	0.17	13.9	0.7	0.14	11.2	0.6	0.11

Table 9
PM exposure: with and without control measures for solid block laying task.

% of PM reduction	Without control measures (mg/m³)			With control measures (mg/m³)								
				70 % efficiency			75 % efficiency			80 % efficiency		
Cumulative PM exposure of workers	PM_{10}	$PM_{2.5}$	PM_1	PM_{10}	$PM_{2.5}$	PM_1	PM_{10}	$PM_{2.5}$	PM_1	PM_{10}	$PM_{2.5}$	PM_1
1	10.9	1.6	0.46	3.2	0.4	0.14	2.7	0.4	0.11	2.1	0.3	0.09
2	9.7	1.6	0.44	2.9	0.4	0.13	2.4	0.4	0.11	1.9	0.3	0.09
3	7.5	1.8	0.44	2.2	0.5	0.13	1.8	0.4	0.11	1.5	0.3	0.09
4	3.4	1.4	0.37	1.0	0.4	0.11	0.8	0.3	0.09	0.6	0.2	0.07
5	9.2	1.6	0.51	2.7	0.5	0.15	2.3	0.4	0.09	1.8	0.3	0.10
6	8.5	1.6	0.43	2.5	0.4	0.13	2.1	0.4	0.13	1.7	0.3	0.07
7	7.3	1.4	0.35	2.2	0.4	0.10	1.8	0.3	0.09	1.4	0.2	0.07
8	2.7	1.4	0.48	0.8	0.4	0.14	0.6	0.3	0.12	0.5	0.2	0.10
9	7.4	1.2	0.44	2.2	0.3	0.13	1.8	0.3	0.11	1.5	0.2	0.09
10	9.5	1.3	0.45	2.8	0.4	0.14	2.4	0.3	0.11	1.9	0.2	0.09
Avg. exposure	7.6	1.5	0.44	2.3	0.4	0.13	1.9	0.3	0.11	1.5	0.2	0.09

sensor showed a 0.80 correlation with the Kanomax PM_{10} monitor and more than 0.90 correlation with the Alphasense sensor. Thus, all the monitoring devices used in the experiment recorded the same static and dynamic trend, as showed in the earlier figures. The amount of respirable and fine particles produced and propagated from a construction can vary from one task to another. The change particle concentration measured during the experiment shows that the ratios of PM_{10} to $PM_{2.5}$ and $PM_{2.5}$ to PM_1 was 18.63, and 6.00 for mixing task and 5.10, and 3.41 for solid block laying task, respectively. The ratios imply that the production of PM_{10} sized particles decreased in solid block laying task compared to the mixing task (i.e., laying activity produced more fine and ultra-fine particles than PM_{10}).

RCS exposure calculated from the mixing task of the activity was higher than the optimum RCS exposure standards proposed by different countries (GESTIS International Limit Values, 2018). According to Stacey et al. (2018) and USEPA (1999), who have defined the RCS concentration in the percentage of the PM_{10} concentration, the RCS concentration is four-fold and two-fold higher than the standard, respectively. As illustrated in Figs. 4 and 6 the exposure to respirable, fine, and ultra-fine particles remain substantially higher even during the erection of solid concrete block wall. The reason for this can be the propagation and behavior of particles of different size and their settlement time. The smaller the size of the particle, the more time it requires to settle down.

The danger of occupational health issues in the construction industry increases as the PM propagates to the breathing area of different skilled construction workers even if they are not conducting activities that produce the PM. To understand the dispersion of different sizes of PM, more factors need to be considered, which can help in future research work. Further experiments are required to analyze the dust exposure from the source at multiple horizontal and vertical distances. Such experiments will help to understand the propagation of the PM (i.e., parabolic or exponential pattern of movement from the source point).

The experimental results expounded by the authors highlight the occupational hazards linked to the construction activity even if the duration is less than an hour. The inhalation of the fine, ultra-fine, and RCS particles can increase the risk of respiratory diseases and also cause premature deaths (Apte et al., 2018). Thus an in-depth assessment of PM emission from the construction work needs to be conducted, to produce a PM inventory for the construction activities. If such a PM inventory is successfully generated by accommodating key parameters like the materials and tools used, type of activity, and duration of activity with and without dust control measures, it can advocate for much sustainable construction production.

A Bio-breathing sensor and a GPS module can be utilized to provide much more specific information regarding the PM exposure of a construction practitioner. Thus, it can help in formulating the cumulative exposure of a construction practitioner who maneuvers through the construction site during a working day. By utilizing such a state of the art techniques with quantification of PM concentration from various construction activities, PM exposure associated with different job roles can be assessed. While quantifying PM generated from different construction activities, the materials and tools used, type of the activity, duration of the activity, and skill level of the worker should be considered. Creating a PM inventory for the construction activities can be an achievable milestone while moving towards a PM prediction model for the construction tasks and activities.

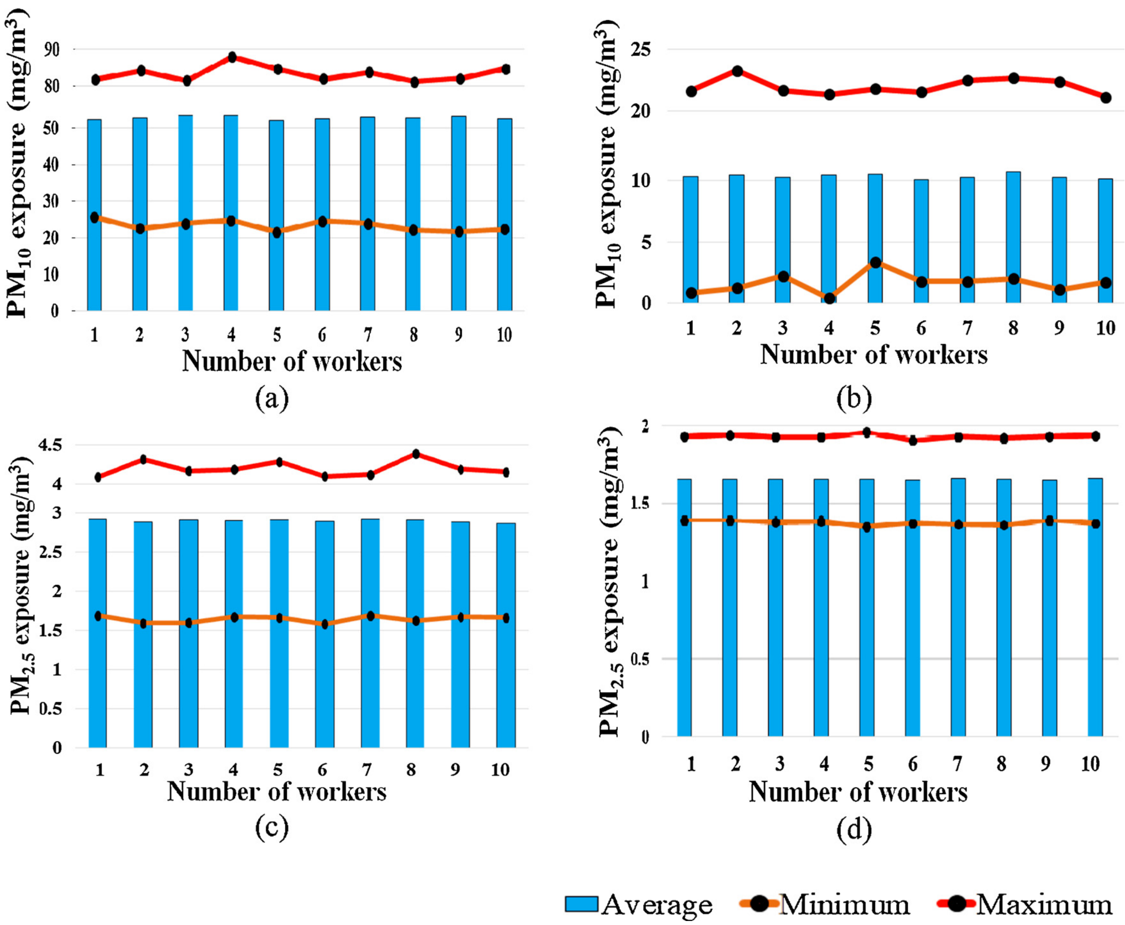

Fig. 9. Average minimum, and maximum exposure from MCS (a) PM_{10} during the mixing task, (b) PM_{10} during the laying task, (c) $PM_{2.5}$ during the mixing task, (d) $PM_{2.5}$ during the laying task.

6. Conclusion

In this research article, the authors have conducted a systematic approach to monitor and measure the PM concentration produced during a construction activity by utilizing modern technological platforms. The execution of the experiment took 40-min for completion (4 min for initial arrangements and conveying materials required for the mixing activity, 16-min for cement-sand mortar mixing, and 20-min for laying the solid concrete block wall). The PM exposure during the preparatory work prior to the mixing task indicates that the material conveyance increases the PM concentration in the working ambiance gradually above the optimum exposure standards within a short duration. The average concentration of PM_{10}, $PM_{2.5}$, and PM_1 during the mixing task were 5330.20, 352.98, and 68.95 μg/m^3 with a σ of 1407.95, 52.26, and 8.22 μg/m^3, respectively. Similarly, the PM concentration recorded during the solid block wall laying task was 1071.30, 175.85, and 49.41 μg/m^3 with a σ of 582.00, 12.7, and 3.84 μg/m^3, respectively. The results of two-stage MCS illustrate that the concentration of PM during the construction activity of 40-min is more than 100 times higher than the PM_{10} exposure standard proposed by WHO and 30 times higher considering the implementation of an efficient control measure.

Limitations of this study can be stated as the lack of PM measurements without control measures, the saturation limit of the Sharp dust sensor, and the accuracy of countermeasures used to reduce particle deflection, as it may vary with distance provided between source and MS. The lack of information about the variation of PM emission in different sized construction elements can also be another limitation of this study. Thus, the reduction of PM concentration due to the implementation of such countermeasures are unknown. Thence, further research in this area can assess many other common construction activities and understand the behavior of PM produced during multiple construction tasks with and without control measures. Thus it may enable the construction practitioners to create much-targeted countermeasures for different construction activities based on its PM emission and to resolve the concerns raised by PM exposure on a macro-scale, dust sensor network can be deployed. It can collect real-time PM concentrations from various construction activities and reduce the cost associated while implementing the same using low-cost dust sensors. The collated information can be stored in a repository for creating a much-targeted control measure for the construction activities.

Declaration of Competing Interest

The authors whose names are listed immediately below certify that they have NO affiliations with or involvement in any organization or entity with any financial interest (such as honoraria; educational grants; participation in speakers' bureaus; membership, employment, consultancies, stock ownership, or other equity interest; and expert testimony or patent-licensing arrangements), or non-financial interest (such as personal or professional relationships, affiliations, knowledge or beliefs) in the subject matter or materials discussed in this manuscript.

Acknowledgments

This work was supported and funded by the Basic Science Research Program through the National Research Foundation of Korea (NRF)

funded by the Ministry of Education (2016R1A6A1A03012812).

References

Alphasense OPC-N2 (2015). *Alphasense user manual OPC-N2 optical particle counter issue 3*. Available at: www.alphasense.com (Accessed 5 March 2019).

Apte, J., et al. (2018). Ambient PM2.5 reduces global and regional life expectancy. *Environmental Science & Technology Letters, 2018*. https://doi.org/10.1021/acs.estlett.8b00360 (Accessed 5 March 2019).

Awolusi, I., et al. (2018). Wearable technology for personalized construction safety monitoring and trending: Review of applicable devices. *Automation in Construction, 85*, 96–106.

Azarmi, F., et al. (2016). Assessment of the long-term impacts of PM10 and PM2.5 particles from construction works on surrounding areas. *Environmental Science: Processes and Impacts, 18*(2), 208–221. https://doi.org/10.1039/c5em00549c Royal Society of Chemistry.

Baron, P. (2010). *Generation and behavior of airborne particles (Aerosols)*. National Institute for Occupational Safety and Health Centers for Disease Control and Prevention.

Beaudry, C., et al. (2013). Occupational exposure to silica in construction workers: A literature-based exposure database. *Journal of Occupational and Environmental Hygiene,* 71–77. https://doi.org/10.1080/15459624.2012.747399.

Budde, M., et al. (2013). Enabling low-cost particulate matter measurement for participatory sensing scenarios. *Proceedings of the 12th international conference on mobile and ubiquitous multimedia - (MUM)' 13,* 1–10. https://doi.org/10.1145/2541831.2541859.

Cao, J., et al. (2013). Evolution of PM2.5 measurements and standards in the US and future perspectives for China particulate matter supersites program view project monitoring and analysis of atmospheric VOCs view project evolution of PM 2.5 measurements and standards in the U.S. *Aerosol and Air Quality Research, 13*, 1197–1211. https://doi.org/10.4209/aaqr.2012.11.0302.

Cao, P., & Zhang, B. (2019). Research and implementation of suppression method of dust pollution environment in large-scale construction. *Ekoloji, 28*(107), 2813–2823.

Cheriyan, D., & Choi, J. (2020). A review of research on particulate matter pollution in the construction industry. *Journal of Cleaner Production, 254*, 120077.

Curto, et al. (2018). Performance of low-cost monitors to assess household air pollution. *Environmental Research, 163*, 53–63.

EN 12341 (1999). *Guidance to member states on PM10 monitoring and intercomparisons with the reference method, group.* 1–69.

EN14907 (2005). *Standardization mandate to CEN, CENELEC and ETSI in support of the implementation of the ambient air quality legislation.* https://doi.org/10.1093/oxfordhb/9780199546282.013.0024.

EPA Air Sensor Guidebook (2014). *Air sensor guidebook, EPA/600/R-14/159*, *1*, 1–5. https://doi.org/10.1017/CBO9781107415324.004.

Fisher, J., Friesen, M., Kim, S., Locke, S., Kefelegn, Y., Wong, J., et al. (2019). Sources of variability in real-time monitoring data for fine particulate matter: Comparability of three wearable monitors in an urban setting. *Environmental Science & Technology Letters, 6*(4), 222–227.

GESTIS International Limit Values (2018). *GESTIS international limit values.* Available at: http://limitvalue.ifa.dguv.de/WebForm_ueliste2.aspx (Accessed 19 March 2019).

Hall, S., et al. (2014). Integrating sensor monitoring technology into the current air pollution regulatory support paradigm practical considerations'. *American Journal of Environmental Engineering, 4*(6), 147–154. https://doi.org/10.5923/j.ajee.20140406.02.

Health and Safety Comission (2005). *Table 1: List of approved workplace'.* 9–25 Available at: http://www.chemeng.upatras.gr/sites/default/files/users/cmngpclab/health-safety/04ENG.ListOfApprovedWorkplaceExposureLimits.pdf (Accessed 10 December 2018).

HSE (2018). *EH40/2005 workplace exposure limits.* Available at: https://books.hse.gov.uk/ (Accessed: 19 March 2019).

Kanomax 3443. Available at: https://www.kanomax-usa.com/product/digital-dust-monitor-model-3443/ (Accessed: 08 Feb 2020).

Khadem, I., & Sgarciu, V. (2014). Smart sensor nodes for airborne particulate concentration detection. *UPB Scientific Bulletin, Series C: Electrical Engineering, 76*(4), 3–12.

Lewis, A., Schneidemsser, E., & Peltier, R. (2018). *Low-cost sensors for the measurement of atmospheric composition: Overview of topic and future applications.* Geneva: World Meteorological Organization4–20. (Accessed by: 07/ 2019) https://www.ccacoalition.org/en/resources/low-cost-sensors-measurement-atmospheric-composition-overview-topic-and-future.

Li, C., et al. (2019). Investigation of dust exposure and control practices in the construction industry: Implications for cleaner production. *Journal of Cleaner Production*. https://doi.org/10.1016/j.jclepro.2019.04.174 Elsevier Ltd.

Li, J., et al. (2018). Spatiotemporal distribution of indoor particulate matter concentration with a low-cost sensor network. *Building and Environment, 127*(November 2018), 138–147. https://doi.org/10.1016/j.buildenv.2017.11.001 Elsevier.

Liu, X., et al. (2016). Low cost sensor network for indoor air quality monitoring in residential houses: Lab and indoor tests of two PM sensors, healthy housing 2016. *Proceedings of the 7th international conference on energy and environment of residential buildings*. https://doi.org/10.4225/50/581072c1872ef (November 2016).

Morgan, G., & Henrion, M. (1992). *Uncertainty: A guide to dealing with uncertainty in quantitative risk and policy analysis.* Cambridge Universirty Press203–207.

Muleski, G., et al. (2005). Particulate emissions from construction activities. *Journal of the Air & Waste Management Association, 55*(6), 772–783. https://doi.org/10.1080/10473289.2005.10464669.

Naticchia, B., et al. (2014). Preliminary tests on a wireless sensor network for pervasive dust moni-toring in construction sites. *The Open Environmental Engineering Journal,* 10–18.

Nij, E., et al. (2003). Dust control measures in the construction industry. *The Annals of Occupational Hygiene, 47*(3), 211–218. https://doi.org/10.1093/annhyg/meg023.

Olivares, G., & Edwards, S. (2015). The Outdoor Dust Information Node (ODIN) – Development and performance assessment of a low cost ambient dust sensor. *Atmospheric Measurement Techniques Discussions, 8*(7), 7511–7533. https://doi.org/10.5194/amtd-8-7511-2015.

Oliveira, M., Izquierdo, M., Querol, X., Lieberman, R., Saikia, B., & Silva, L. (2019). Nanoparticles from construction wastes: A problem to health and the environment. *Journal of Cleaner Production, 219*, 236–243.

OSHA (2018). *Crystalline silica.* (Accessed by: 02/2020) https://www.osha.gov/dsg/annotated-pels/tablez-1.html.

Patel, et al. (2016). Spatio-temporal measurement of indoor particulate matter concentrations using a wireless network of low-cost sensors in households using solid fuels. *Environmental Research, 152*, 59–65.

Peters, S., et al. (2009). Personal exposure to inhalable cement dust among construction workers. *Journal of Environmental Monitoring, 11*(1), 174–180. https://doi.org/10.1039/B812357H Royal Society of Chemistry.

SCAQMD (2018). *The south coast air quality management district.* Available at: http://www.aqmd.gov/aq-spec/evaluations/summary (Accessed: 11 July 2018).

Sharp Corporation (2006). *Sharp corporation.* Available at:http://www.sharp-world.com/products/device/lineup/data/pdf/datasheet/gp2y1010au_appl_e.pdf.

Sousan, S., et al. (2016). Evaluation of the alphasense optical particle counter (OPC-N2) and the grimm portable aerosol spectrometer (PAS-1.108). *Aerosol Science and Technology, 50*(12), 1352–1365. https://doi.org/10.1080/02786826.2016.1232859.

Stacey, P., et al. (2018). Determination of respirable-sized crystalline silica in different ambient environments in the United Kingdom with a mobile high flow rate sampler utilising porous foams to achieve the required particle size selection. *Atmospheric Environment, 182*(March), 51–57. https://doi.org/10.1016/j.atmosenv.2018.03.032 Elsevier.

The Community Robotics (2019). *The community robotics.* Available at: http://explorables.cmucreatelab.org/explorables/air-quality-monitor-tests/#data (Accessed 11 July 2018).

Tian, G., Wang, J., Lu, Z., Wang, H., Zhang, W., Ding, W., et al. (2019). Indirect effect of PM1 on endothelial cells via inducing the release of respiratory inflammatory cytokines. *Toxicology in Vitro, 57*, 203–210.

Tong, R., et al. (2018). The construction dust-induced occupational health risk using Monte-Carlo simulation. *Journal of Cleaner Production, 184*, 598–608.

United Nations - Department of economic and social affairs (2014). *World urbanization prospects. The 2014 revision.* ST/ESA/SER.A/35232.

US EPA (2018). *Evaluation of emerging air pollution sensor performance.* Available at: https://www.epa.gov/air-sensor-toolbox/evaluation-emerging-air-pollution-sensor-performance (Accessed 11 July 2018).

USEPA (1999). *Estimating particulate matter emissions from construction operations. Final report*Kansas: Eastern Research Group.

Webster, G. (1999). *The measurement, instrumentation, and sensors handbook.* CRC Press published in cooperation with IEEE Press.

WHO (2005). *Air quality guidelines. Global update 2005. Particulate matter, ozone, nitrogen dioxide and sulfur dioxide.* https://doi.org/10.1007/BF02986808.

Wu, Z., et al. (2016). Mitigating construction dust pollution: State of the art and the way forward. *Journal of Cleaner Production, 112*, 1658–1666. https://doi.org/10.1016/j.jclepro.2015.01.015 Elsevier Ltd.
